# Brain Mechanisms and Reading Remediation: More Questions Than Answers

**DOI:** 10.1155/2014/802741

**Published:** 2014-01-12

**Authors:** Kristen Pammer

**Affiliations:** The Department of Psychology, The Australian National University, Canberra, ACT 0200, Australia

## Abstract

Dyslexia is generally diagnosed in childhood and is characterised by poor literacy skills with associated phonological and perceptual problems. Compensated dyslexic readers are adult readers who have a documented history of childhood dyslexia but as adults can read and comprehend written text well. Uncompensated dyslexic readers are adults who similarly have a documented history of reading impairment but remain functionally reading-impaired all their lives. There is little understanding of the neurophysiological basis for how or why some children become compensated, while others do not, and there is little knowledge about neurophysiological changes that occur with remedial programs for reading disability. This paper will review research looking at reading remediation, particularly in the context of the underlying neurophysiology.

## 1. Brain Mechanisms and Reading Remediation: More Questions Than Answers 

Approximately 10% of children suffer a specific reading difficulty such as dyslexia [1]. Despite some residual deficits in core skills, (e.g., phonological processing), some of these individuals will ultimately learn good reading skills as adults (become compensated), while others will remain functionally reading-impaired all their lives (uncompensated) [2, 3]. On the last page of her seminal book on dyslexia, Snowling [2] concludes “*The research agenda for the next decade must certainly be directed to the treatment resisters,… those poor readers who do not respond well to current intervention programs*.” Yet despite the huge personal and social costs of dyslexia, virtually nothing is known about how or why some young dyslexic readers ultimately learn to read, while others remain functionally dyslexic their whole lives. The aim of the current review is to consider some of the research on reading remediation, particularly within the context of underlying brain mechanisms.

A number of reviews have been conducted regarding the functional organisation of the normal reading network in the brain (refer to [4] for a recent review) and there is some research that has looked at compensatory brain mechanisms that develop as poor readers develop good reading skills [5]. However, a full understanding of how cortical networks develop in response to acquiring reading skills requires not only an understanding of what those networks look like but also an understanding of how those networks are functionally connected. Functional connectivity in language is well documented (e.g., [6]), and it is common for researchers to draw on this literature to also describe reading networks; however, cortical networks in reading are quite different from cortical networks in language. Our ability to acquire and use language is entirely different from our ability to learn to read and very different from the processes involved in reading fluently. The primary difference is that language acquisition and use are likely to be innate, whereas reading skills are learned. This is an important difference because the former assumes the existence of a naturally occurring underlying biological substrate, whereas the latter does not. When reading the brain has had to learn to recruit resources from quite disparate parts of the brain that have likely evolved to perform quite unique functions. Therefore, reading is a learned process that is quite different from the acquisition of language which is more like fine tuning preexisting neural circuitry.

It is assumed that lexical representations are encapsulated as specific neural traces, and the various components of the reading process, such as pattern recognition, and memory, phonological decoding, are similarly represented as neural responses in the brain. When reading, the brain has had to learn to recruit resources from quite disparate parts of the brain that have likely evolved to perform quite unique functions. Thus, with all learned skills, there are some individuals who are good at the skill, many who are average, and a number who are very poor. In reading, the very poor category constitutes the group of children that we consider to be dyslexic.

The following review is designed to consider theoretical models for ways in which the brain might compensate for dyslexia and to evaluate some of the scientific knowledge in this area. The aim is to broaden and clarify our understanding of the dynamics and cortical plasticity involved in acquiring reading skills. Then at the practical level, if we can understand how compensated dyslexics read, we can make predictions about how they overcame their disability and provide a strong scientific foundation from which to later formulate strategic intervention programs to facilitate successful reading skills in all dyslexic readers. Finally, an understanding of these concepts has broader implications for neuroscience, by contributing to our understanding of neural plasticity in large, distributed cognitive networks.

## 2. Dyslexia Sometimes Persists into Adulthood

Dyslexia is not just a childhood problem. Many adults who have suffered from developmental dyslexia as children never develop good reading skills [7]. Those who do compensate for their reading difficulty and become good readers invariably suffer from residual difficulties such as poor spelling and poor phonological coding [8]. Uncompensated dyslexic readers never develop functional reading skills despite normal exposure to reading instruction, and in some cases many hours of reading remediation. These readers are the treatment resisters [2].

Compensated dyslexic readers—also sometimes referred to as resilient readers [9]—are adult readers who have a documented history of childhood dyslexia but as adults can comprehend written text well, in spite of residual difficulties in low-level decoding skills [3, 10–12]. These are adult readers who are functionally sound readers and are quite often high achievers, despite having a history of childhood reading difficulties. Little is known about how dyslexic children go on to develop reasonable reading skills; much of the literature suggests that such children develop idiosyncratic behavioural coping strategies as a consequence of high levels of motivation and educational opportunities (e.g., [13]). Nevertheless, the question of interest here is, *neurophysiologically*, how is it that a dyslexic child has learned to read? Has the brain adapted to the reading difficulty and slowly developed a normal reading network? Or has the brain developed a new, compensatory network to bypass functional impairment?

Conversely, there are adult dyslexic readers who similarly have a documented history of childhood reading impairment, but unlike compensated dyslexics, they remain functionally reading impaired all their lives. Such adults will still score well below average on standard adult reading tests, despite normal adult IQ and—again—no comorbid difficulties. These are referred to as uncompensated dyslexic adults (or sometimes adults with persistent dyslexia), as their poor reading and spelling skills have persisted into adulthood.

Neurophysiologically, compensated and uncompensated dyslexic adults are likely to be quite distinct: in one case the adult brain has adapted to a childhood, developmental impairment to learn the skill of reading, whereas in the other, the brain has not developed a functional reading network at all or has developed a reading network that remains dysfunctional, supporting only rudimentary reading skills. Few studies have explored the neurophysiological distinction between the two groups. More importantly, there is little consistent convention in the scientific literature of the use of either group, such that many—if not most—studies using adult dyslexics fail to report whether the participants are compensated or not. Clearly, this can be a significant problem given that the neurophysiological profiles of each of the groups are likely to be quite different, making it difficult to make unambiguous statements about cortical functionality in adult dyslexics.

As an aside, it is worth noting that both compensated and uncompensated dyslexia in adults are different from acquired dyslexia which is also usually associated with adults. In the former, poor reading skills have been a life-long affliction. However, in the latter, the adult has had a history of normal reading as a child, but reading difficulties occur as a consequence of specific brain injury such as stroke or an accident. Here then, neurophysiologically, reading difficulties are reasonably straight forward and easily identified as a consequence of specific damage to a specific part of the cortex. Referred to as Alexia, it seems to be associated with left occipital lesions with right homonymous hemenopia (e.g., [14–17]), although pure alexia has also been associated with thalamic impairment [18], without homonymous hemenopia (e.g., [19, 20]), and closer to the inferior temporal areas [21]. The most common understanding of the disorder is as a disconnection syndrome such that bilateral visual stimuli fail to reach the angular gyrus—as described by Déjerine back in 1891 [22].

## 3. The Neurophysiology of Component Behavioural Mechanisms in Compensated Dyslexia

In order to understand the cortical maps involved in reading and dyslexia, it is important to become familiar with the component neurocognitive mechanisms of the network. There are a number of behavioural mechanisms that have been implicated in dyslexia.

### 3.1. Sensory Processing

There is a large collection of evidence implicating early sensory coding difficulties in dyslexia in the visual domain [23–33] and auditory processing [34–37]. However, the degree to which sensory coding persists into adulthood and can characterise compensated and uncompensated adult readers remains unclear. Birch and Chase [38] failed to find differences on measures of visual processing such as contrast sensitivity and sine wave detection, although they used static stimuli. Hämäläinen et al. [39] measured rise times in amplitude modulation in auditory processing. Again there were no systematic differences between compensated and uncompensated groups, but there was a correlation between phonological ability and sensitivity to rise times, which may explain the lack of difference between the groups, as poor phonological sensitivity in dyslexic readers frequently persists into adulthood irrespective of reading skill [12, 40, 41].

### 3.2. Phonological and Orthographic Processing

Despite the acquisition of normal functional reading skills, compensated dyslexic readers frequently maintain residual problems in phonemic awareness [3, 10, 12]. Given that regular words can be read by either conversion of graphemes to phonemes, or by the recognition of the orthographic form of a word, it has thus been suggested that normal reading skills in compensated readers are acquired as a result of dependence on whole-word orthographic skills [42]. Neurophysiologically, compensated dyslexic readers engage in different cognitive networks when processing tasks that require phonological manipulations, such as less activation for compensated readers in the insula, left premotor, and Wernicke's regions [43]. A study directly comparing normal, compensated, and uncompensated readers on a nonword rhyming task found that both dyslexic groups demonstrated less activation in superior-temporal and occipitotemporal regions with overactivation in right inferior frontal areas. Compensated readers differed from both groups by activating right superior frontal and mid-temporal regions [44]. Ingvar et al. [45] investigated differences between normal and compensated dyslexic readers in a single word reading task, demonstrating that compensated readers showed an increased activation in right temporal regions.

### 3.3. Semantic Encoding

There is substantial evidence for a dissociation between word decoding and semantic processing or comprehension [46]. Some evidence suggests that both adult dyslexic readers [47] and children with dyslexia [48, 49] may rely more heavily on the influence of semantic context when reading, with research implicating stronger activation in the inferior frontal regions when processing semantic and contextual information [50]. Similarly, underengagement of the dorsal and ventral aspects of the left posterior cortex has been demonstrated in poor readers, but with a disproportionately higher activation in the inferior frontal gyrus (IFG), suggested to be important in the integration of semantic associations [47, 51, 52]. Moreover, distributed models of reading comprehension have suggested that poor low-level decoding skills may be compensated by more sophisticated higher-level skills such as semantic context [53]. Again therefore, while some studies have suggested that compensated readers may recruit from different areas of the network to enable greater reliance on semantic encoding, few studies have investigated this in the context of adult reading skills ranging from compensated to uncompensated. Thus, we do not know if this is a successful adaptation acquired by better readers or a general adaptation arising from reading failure.

## 4. How Do Poor Readers Become Compensated?

Neurophysiologically, there are a number of ways in which a child with dyslexia could develop functionally adequate reading skills. There is little evidence supporting any position, and the following are logical derivations from our knowledge of existing reading networks and how complex cortical connectivity occurs. Thus the following are hypotheses, exploring these should be the focus of the next generation of research.

One possibility is that dyslexic readers ultimately learn good reading skills (become compensated readers) by eventually developing the cortical connections required for normal reading networks rather than developing uniquely different cortical connections. The assumption here then is that normal reading is associated with the development of consistent cortical networks that are observable over most—if not all—readers. That there is a normal reading network that is consistent over most readers has been demonstrated elsewhere. For example, we demonstrated [54] that unique areas of the brain synchronised at 8–12 Hz (alpha range) in response to different reading requirements. In this study, participants were presented with continuous text presented at rates that made comprehension easy, effortful, very difficult (only the general gist of the story was apparent), or impossible (random text). Left hemisphere cortical activations consistent with a reading network were activated at 8–12 Hz in a dynamic way that reflected the cognitive requirements of the reading task and were consistent over participants.

The scenario that adult dyslexics simply develop the normal networks eventually points to the possibility that dyslexia occurs as a consequence of slow or delayed development of dedicated reading networks. If this is the case, then a logical biological substrate would be that this occurs as a result of poor or slower neuronal maturation. This is consistent with Wright and Zecker [55] who found age-dependent differences in auditory functioning for dyslexic children, and they suggested that slower neurological development may be further arrested with the onset of puberty. Certainly, it is well accepted by neurophysiologists that neuronal plasticity decreases in older animals that have reached sexual maturity [56]. Moreover, McArthur and Bishop [57–60] have put forth the Maturational Hypothesis, where dyslexia may be partially caused by delays in the development of cortical connectivity rather than a specific deficit in the network itself. Thus, compensation in this neurophysiological scenario occurs because a normal, predictable neural network eventually develops as the neural connections strengthen.

Presumably then, persistent adult dyslexia is a consequence of the normal network failing to reach full maturity. This proposal points to clear empirical predictions: using neuroimaging techniques, it should be possible to demonstrate that all adult readers, both normal and dyslexic, should show activation in the same basic regions in the same order and demonstrate the same connectivity. Poorer adult readers would show weaker activation and/or temporally delayed (slower) signals [61]. This would show up as a positive correlation for reading ability with activation and a negative correlation between latency and reading ability.

Another possibility is that dyslexic readers become compensated readers because they develop new, alternative cortical connections to support reading that are unique to the individual. Here then, there is a normal reading network observable in most readers, but for some reason in dyslexic readers this network fails to develop, but with constant exposure to reading, the individual develops a bypass model to support reading skills. Because learning the skill of reading ultimately requires the development of new cortical networks for all readers, there is no specific reason that the brain must solve the same problem (learning to read) in exactly the same way for all people. It is not unreasonable to expect that if the cortical connections that develop to support reading are unsuitable for whatever reason, then through remediation, the brain could develop entirely different connections to support the same behavioural outcome (reading). There is some support for this proposition as well, with the evidence that some dyslexic readers activate unique areas when reading compared to normal readers. For example, there is a tendency for poor readers to demonstrate abnormal activations in the left temporoparietal regions of the brain during language processing and reading tasks [51, 62]. Pugh et al. [47] have suggested that when reading, poor readers engage frontal sites, such as the IFG and prefrontal dorsolateral sites [52, 63, 64], more so than normal readers. Similarly, Salmelin et al. [61] and Brunswick et al. [51] showed greater activation of inferior precentral gyrus (Broca's area) in poor readers when processing visually presented words with post-200msec responses. Thus, for some reason (e.g., poor sensory input), the normal reading network cannot be used and neuronal patterns of activation attempt to bypass the deficient mechanisms to meet reading requirements. Indeed, there is also evidence of white-matter connectivity differences between dyslexic and normal readers [65]. Furthermore, Horwitz et al. [66] demonstrated that compared to control adult readers, compensated adult readers failed to activate the same brain areas during reading tasks. In this study they measured the functional correlation of activity across the brain with the left angular gyrus to reading irregular and nonwords. They demonstrated that normal readers generated an activity pattern that included visual association areas and Wernicke's area; however, such a pattern of activity was absent in the compensated dyslexics. See also Wimmer et al. [67] who demonstrated different patterns of activity in compensated dyslexic adults compared to normal readers, particularly in visual, occipital areas.

**Figure 1:**
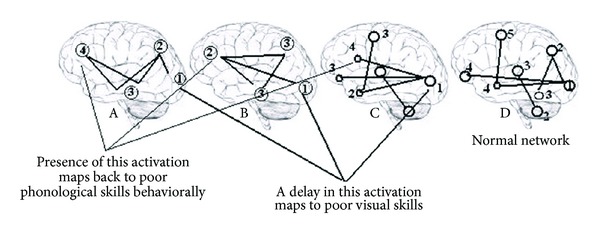
Individual dyslexic readers (brains A, B, C) may develop their own unique cortical networks, but common deficits map back onto common behavioural deficits. Brain D here is from Kujala et al. [54] representing normal network connectivity when reading.

If a new, unique reading network develops, then logically there are a number of ways in which this could occur: once the normal reading network is unattainable, there is another common network that is accessed to meet reading requirements, irrespective of the type of damage existing in the normal system. Compensated dyslexic readers simply get better at using this network than uncompensated readers. This would predict that the network dynamics for dyslexic readers would be different from good readers, but consistent within a dyslexic group. Another possibility is that different spatiotemporal maps of activity develop to bypass particular damage. For example, an increase in activity in the IFG may be associated with poor phonological skills as found by Pugh et al. In this scenario, similar neural connections that develop in response to reading would map onto similar behavioural results in component reading skills. Common components of the network (e.g., increased activation in the IFG) in different reading situations would be associated behaviourally with component skills (e.g., poor phonological sensitivity). Compensatory mechanisms may develop in this case with the need for fewer bypass mechanisms or the more efficient use of the mechanisms. In another possibility, dyslexic readers in general develop unique and idiosyncratic neurophysiological mechanisms to read. Here then, compensatory behaviour occurs as a result of the development of more successful connections that is unique to the individual and predicated on other factors such as motivation and individual strategy development. In this scenario, there would be little systematic mapping to behavioural outcomes. Some examples are described in Figure 1.

Thus, there are a number of logically derived possibilities to explain how compensatory mechanisms might develop, and the strength of these possibilities is that they generate clear and testable hypotheses which should be the focus of the next wave of research into dyslexia and reading remediation.

## 5. Changes in Cortical Connectivity When Dyslexic Readers Learn to Read

Some research has been conducted looking at neurophysiological changes in children after remedial programs. It may be possible to extrapolate from these studies to support one or more of the proposals suggested previously.

Reading improvement in children with dyslexia has been demonstrated to be associated with activity in the left inferior frontal gyrus [68] such that white matter integrity was positively correlated with reading gain. This indicates that those dyslexic children who are most likely to acquire good reading skills are those children who have more extensive connectivity in the left inferior frontal cortex. Thus, connectivity with the right inferior frontal cortex may be an important component in the development of compensatory brain networks in reading. This is consistent with other studies looking at the development of compensatory mechanisms (e.g., [69–72]).

Structural changes in the brain have been well documented as a consequence of reading intervention and typically involve phonological interventions. For example, Eden et al. [70] tested adult dyslexic and nondyslexic readers before, and then after eight weeks of a multisensory, phonological-based reading intervention. They demonstrated an increase in response in the left angular gyrus, and the fusiform/parahippocampal gyrus anterior to the Visual Word Form Area, in response to phonological tasks. Changes in white and grey matter have also been demonstrated: Keller and Just [73] investigated white matter organisation in dyslexic children after 100 hours of phonological reading and spelling instruction. After training, the children performed better at phonological tasks, and this was correlated with an increase in white matter—specifically in left anterior tracts. Indeed, the location of increased white matter was the same area that showed decreased activation in dyslexic readers compared to good readers before intervention. Grey matter changes have also been demonstrated with intervention. Krafnick et al. [74] demonstrated increases in grey matter volume in the left fusiform and precuneus and right hippocampus and cerebellum. Interestingly, the intervention used here was less phonological and was based more heavily on imagery and multisensory processing. Given the multitude of reading intervention possibilities, it remains to be seen whether the type of reading intervention is important for neurophysiological changes in response to reading intervention.

That behavioural change reflects changes in cortical connections is highlighted in a recent paper by Koyama et al. [5]. These authors employed a technique called intrinsic functional MRI (I-fMRI). This technique is unique in that it represents a way of measuring functional connectivity between different brain regions when at rest [75]. By identifying a specific brain region, researchers are able to identify other parts of the brain that demonstrate correlated fluctuations in activity over time. This provides an excellent mechanism for understanding and mapping large-scale cortical interactions that are particularly characteristic of cognitive functioning (refer to [76] for a recent review) because it turns out that many of the neural networks that are activated when an individual engages in a cognitive task, are also active when the brain is at rest. This is a particularly useful technique for investigating cognitive disorders such as dyslexia, because the participant does not need to engage in any reading behaviour, thus avoiding many of the other confounds that plague dyslexia research such as performance motivation, stress, and anxiety. Koyama et al. investigated I-fMRI in dyslexic and control children where the dyslexic children were either dyslexic with no intervention, dyslexic but had experienced reading intervention, or dyslexic and had experienced reading and spelling intervention. The interventions in this case varied from child to child but were predominately language based, and all the remediated children had no demonstrable reading problem by the time of the study. Of the many seeding locations initially identified in the brain, the authors demonstrated that intrinsic functional connectivity between the left intraparietal sulcus and left middle frontal gyrus (BA9) was significantly correlated with reading intervention, with normal readers showing the strongest connectivity, followed by the two intervention groups, with the no-intervention dyslexic group showing little or no connectivity. Similarly, the left fusiform gyrus demonstrated differential functional connectivity between the groups. For example, connectivity strength with frontal areas was higher for the two intervention groups but virtually nonexistent for the control and no-intervention groups. This is a particularly exciting study for demonstrating the efficacy of training techniques. Of particular interest is that reading intervention is not only effective in developing normal connectivity, which supports the Neuronal Maturation hypothesis proposed above, but also in developing compensatory connections in the brain. This has enormous implications for the development of reading interventions.

However, the vital piece of the puzzle that is missing here thus far in how dyslexic readers become compensated readers is the link with cortical frequency dynamics. It is not just where cortical connections are formed in the brain that is important, but how the different parts of the brain communicate that is vital for a full understanding of brain activity in dyslexic remediation. This is particularly important when evaluating different models of compensation because timing between activations at cortical sites may be just as important as the spatial distribution (e.g. [30, 31, 61, 77]).

## 6. Frequency Dynamics and Cortical Connectivity

Both EEG and MEG measure the synchronous firing of large populations of cells in the cortex. Cells within a given population are said to be firing synchronously when their firing rhythm coincides at a particular frequency, and different firing frequencies are believed to reflect different functional states.

Changes in oscillatory power refer to an increase or decrease in the amplitude of power within specific frequency bands. It is believed to reflect changes in oscillatory dynamics at the local level [78], potentially within a particular cortical area or structure. Event-related synchronisation (ERS) and event-related desynchronisation (ERD) reflect increases or decreases in power, respectively. When a functionally specific subset or population of neurons process incoming information, they will disengage from the larger neuronal set to synchronise and oscillate at a different frequency.

Oscillatory coherence is believed to reflect the transient synchronisation at specific frequencies of disparate areas of the cortex [78], further than 1 cm or so away from each other [79]. This is the situation where large-scale neuronal assemblies across the cortex start talking to each other. While ERS and ERDs are likely to reflect local functionality in specific cortical sites, oscillatory coherence is more likely to reflect complex cognition, where many different parts of the brain need to talk to each other quickly and fluidly such as in reading.

Different cortical areas exhibit rhythmical activity to internal or external input, with characteristic frequency ranges, such as 8–12 Hz (alpha oscillations), 13–24 Hz (beta oscillations), 25–50 Hz (gamma oscillations), and >50 Hz (high gamma oscillations), and spatially distributed components of cerebral networks are assumed to talk via such synchronised neural firing [80]. Cognitive functions are thought to depend on this type of connectivity between large-scale neural networks [81]. Therefore, the frequencies at which populations of neural cells oscillate are believed to be a mechanism by which different regions of the brain communicate, with different sensory and cognitive experiences inducing unique oscillatory signatures. The functional significance of synchronous oscillations has been demonstrated as mediating other cognitive processes, such as memory [82–85], attention and attentional processes (e.g., [86]), face perception (e.g., [87]), and object detection (e.g., [88–90]).

Consistent with other cognitive processes, it is not unreasonable to predict that reading and word recognition rely heavily on the functional states of cortical networks. However, little research has been conducted to look at connectivity in terms of frequency dynamics between different cortical areas in response to intervention. This is a vital piece of the puzzle when it comes to reading intervention and remediating poor readers, as changes in reading skill may be closely associated with changes in the way in which populations of neurons communicate, rather than just changes in spatial maps of activity. Nazari et al. [91], used neurofeedback to train children to decrease delta (1–4 Hz) and theta (4–8 Hz) brain oscillations and to increase beta (15–18) oscillations over eight training sessions. They demonstrated an increase in reading speed and accuracy with neurofeedback training. However, they did not explicitly manipulate reading intervention, and the dependent variable was reading outcome, although they do also report an increase in coherence for theta rhythms. So the logic of this study was that explicitly decreasing delta and theta rhythms and increasing beta rhythms resulted in improved reading outcomes. However, it is unclear what the behavioural changes are in response to and whether the results could have been due to test repetition.

Although little or no research has looked at cortical frequency dynamics in dyslexia remediation, it has recently been investigated in the context of language impairment, which has been reported to have a 50% comorbidity with dyslexia [92]. In this study [93], children with language impairment were given language remediation, some of which is phonologically based. The neurophysiological responses were measured in response to passive listening to tone pairs. They specifically focused on gamma activity in this study and demonstrated an increase in gamma activity and gamma phase locking for the children who had experienced the intervention. Thus, this recent project provides in-principal evidence for the importance of cortical coherence in reading remediation.

## 7. Conclusion

Understanding the processes by which some dyslexic children come to learn to read as adults provides an enormous resource for understanding the plasticity of complex cognitive networks such as reading, how neurobiological processes can adapt to meet specific cognitive requirements, and how we might better design remedial reading treatment in children to exploit such processes. For some young dyslexic readers it might be better to foster reading skills that already exist rather than attempt to develop functionality in brain mechanisms which are less responsive. Demonstrating successful cortical plasticity in compensated dyslexic adults addresses questions regarding whether treatment intervention should target the relative strengths of the dyslexic reader rather than persisting in standard intervention practices. Such research also has important consequences for how we teach reading; identifying intrinsic functional components of the cortical reading network would provide vital information to inform current debates regarding the relative importance of whole-word versus phonological decoding in reading instruction. By identifying in compensated adults, those components of the neural reading network that are intrinsic to the reading process, such research would provide a basis for strategies for the early detection of dyslexia, such that deficits in component skills could be identified before children learn to read.

Thus, how dyslexic readers develop neural connectivity to ultimately acquire good reading skills remains speculative; however, research such as that of Koyama et al. [94] suggests that the full story may ultimately be a hybrid of the possibilities posited above, with dyslexic readers developing both new and idiosyncratic connections, as well as finally developing normal reading networks. Nevertheless, a major limitation of this recent study is its cross-sectional design. In this it is difficult to make assumptions about the antecedent behaviors that might have occurred before testing. and a lack of ability to control the nature of the intervention. Subsequent studies are now well placed to develop this concept further by using longitudinal designs in which the intervention is targeted and controlled with a larger sample of children.
